# Tetracycline and chloramphenicol exposure induce decreased susceptibility to tigecycline and genetic alterations in AcrAB-TolC efflux pump regulators in *Escherichia coli* and *Klebsiella pneumoniae*

**DOI:** 10.1371/journal.pone.0315847

**Published:** 2025-01-22

**Authors:** Nian Anwar Nasralddin, Mehri Haeili, Sasan Karimzadeh, Fatemeh Alsahlani

**Affiliations:** Department of Animal Biology, Faculty of Natural Sciences, University of Tabriz, Tabriz, Iran; University of Buea, CAMEROON

## Abstract

Tigecycline (Tgc), a third-generation tetracycline is found as the last line of defense against multi-drug resistant bacteria. Recent increased rate of resistance to tgc, a human-restricted agent among animal bacteria poses a significant global health challenge. Overuse of first generation tetracyclines (Tet) and phenicols in animals have been suggested to be associated with Tgc resistance development. In the current study we aimed to determine the effect of tetracycline (Tet) and chloramphenicol (Chl) overexposure on Tgc susceptibility. A Tet and Chl-susceptible isolate of *K*. *pneumonia*e and *E*. *coli* were exposed to successively increasing concentrations of tetracycline and chloramphenicol separately until a ≥4 times increase in Tet and Chl MICs was observed. Susceptibility changes to several antimicrobial agents were tested using disk diffusion and broth dilution methods. The genetic alterations of genes coding for major AcrAB regulators including *acrR* (repressor of *acrAB*), *ramR* (repressor of *ramA*), *soxR* (repressor of *soxS*) in *K*. *pneumoniae* and *lon* (proteolytic degradation of MarA), *marR* (repressor of *marA*), *acrR* and *soxR* in *E*. *coli* were investigated. The expression level of *acrB* was measured using reverse transcription-quantitative polymerase chain reaction (RT-qPCR) method. The excessive exposure (15 to 40 selection cycles) of studied bacteria to both antibiotics significantly decreased susceptibility of Tet-resistant (R) and Chl-R variants of *E*. *coli* (n = 6) and *K*. *pneumoniae* (n = 6) to several groups of antibiotics including tigecycline (4–16 and 8–64 times respectively) and quinolones. About 58% of variants (n = 7) carried genetic alterations in AcrAB regulators including *ramR* (frameshift mutations/locus deletion), MarR (L33R, A70T, G15S amino acid substitutions) and Lon (L630F change, frameshift mutation) which were associated with *acrB* upregulation. Our study demonstrated the capacity of chloramphenicol and tetracycline exposure for selection of mutants which revealed tigecycline resistance/decreased susceptibility mostly mediated by active efflux mechanism. Unaltered *acrB* expression level in some strains indicates possible contribution of other efflux pumps or non-efflux-based mechanisms in the development of multiple- antibiotic resistance phenotype.

## Introduction

The crisis of antimicrobial resistance (AMR) is widespread and still increasing, due to inappropriate use of antibiotics and unavailability of newer agents. According to recent research on the global burden of AMR, more than 4.9 million deaths were associated with AMR in 2019 [1, 2]. Drug-resistant diseases are predicted to cause 10 million deaths a year and 100 trillion USD cumulative global economic cost per year by 2050 [3, 4]. Tigecycline (Tgc), a third-generation tetracycline (Tet) within the glycylcycline class typically serves as a last-resort agent for sever infections caused by medically important multidrug-resistant (MDR) bacteria. Principally, it is used for treatment of critically ill patients infected with colistin and /or carbapenem resistant *Enterobacterales* or *Acinetobacter baumannii* highlighting its crucial role in clinical settings [5–9]. Unlike older generation tetracyclines, Tgc is a human restricted medicine overcoming typical tetracycline resistance mechanisms mediated by Tet-type efflux or ribosomal protection proteins [10]. Despite superior antibacterial activity, Tgc resistance has recently emerged among different members of *Enterobacterales* of human and animal origin as a result of increased clinical reliance to tgc and possibly improper use of first-generation tetracyclines [11, 12]. Tigecycline resistance in carbapenem resistant *Enterobacterales* has serious public health implications that can eventually lead to treatment failure and high mortality rate [12, 13]. The tigecycline resistance mechanisms in *Enterobacterlaes* is not fully understood and is found to be multifactorial. Overexpression of the genes encoding the Resistance-Nodulation-Division (RND)-type efflux pump, AcrAB which confers decreased susceptibility to a wide range of commonly used antibiotics by exporting antibiotics from the cell, constitutes the most common resistance mechanism [14, 15]. The expression of *acrAB* is controlled by several regulators including the local repressor AcrR and global transcriptional factors MarA/RamA and SoxS [16]. Loss-of-function mutations occurring in AcrR and RamR (repressor of *ramA*) have been found to result in overexpression of *acrAB* among Tgc resistant *Klebsiella pneumoniae* (Tgc-R-KP) isolates [17, 18]. In *Escherichia coli*, mutations in MarR (the transcriptional repressor of MarA), and ATP-dependent Lon protease (involved in proteolytic degradation of MarA and SoxS [19]) are reported to contribute to Tgc resistance development [20, 21]. Altogether these mutations result in the relief of transcriptional repression, consequent upregulation of the AcrAB efflux pump and resistance to extruded antibiotics. Mutations in *rpsJ* encoding ribosomal S10 protein, [22] which result in weaker binding of tigecycline to the 16S rRNA (target site-associated resistance mechanism) [23], extrusion of Tgc by a mutated TetA efflux pump [22] and also enzymatic inactivation by *tet*(X) family genes (encoding flavin-dependent monooxygenases) are other factors contributing to reduced susceptibility to Tgc [24].

The agricultural use of human antibiotics in farm animals for disease prevention and also as growth promoters is blamed for contributing to the rising threat of antibiotic resistance as it is believed to encourage the emergence of antibiotic resistant bacterial clones in farm animals that can spread to humans through the food chain, direct contact, or the environment [25]. Therefore, emergence of bacterial clones resistant to clinically important antibiotics such as tigecycline from animal sources poses a great risk to public health. In our recent study, we found a series of Tgc-R-KP isolates from healthy broilers which revealed overexpressed AcrAB efflux pump mainly due to AcrR and RamR alterations. Moreover, we found that Tgc resistance induction in the presence of selective pressure of Tgc, resulted in development of *E*. *coli* and *K*. *pneumoniae* mutants which were not only resistant to Tgc and other members of Tet family but also to chloramphenicol (Chl) [11, 21]. According to this finding and considering the lack of Tgc animal-use, we proposed that massive application of antibiotics other than Tgc itself, such as first-generation tetracyclines and phenicoles (such as florfenicol) in animals or veterinary medicine [26–28] presumably provide driving forces for the selection of Tgc-R clones by inducing AcrAB overexpression through genetic alterations in regulators of this efflux pump. Therefore, in the current study we exposed a tetracycline and chloramphenicol susceptible isolate of *K*. *pneumonia*e and *E*. *coli* to successively increasing concentrations of tetracycline and chloramphenicol separately and, tested the susceptibility of developed variants to Tgc as well as other antimicrobials. Thereafter, genetic alterations of AcrAB regulatory systems and *acrB* expression levels were investigated.

## Materials and methods

### Bacterial isolates

This research included one clinical *E*. *coli* isolate and one animal-derived *K*. *pneumoniae* (obtained from a healthy broiler) isolate. The isolates were obtained during our previous studies and identified using whole genome sequencing (*E*. *coli*, strain EC309wt SRR18110788) or conventional biochemical tests (*K*. *pneumoniae*) including IM-ViC tests (indole test, methyl red test, Voges-Proskauer reaction, citrate utilization test), motility, reactions observed on Triple Sugar Iron (TSI) agar (H2S and gas production, carbohydrate utilization pattern), urease and ONPG (O-nitrophenyl-beta-D-galacto- pyranoside) tests [29]. Both isolates were selected based on their phenotypic susceptibility to tetracycline (minimum inhibitory concentration (MIC) = 4 mg/L) and chloramphenicol (MIC = 4 or 8 mg/L).

### *In vitro* selection of tetracycline and chloramphenicol resistant mutants

In order to obtain laboratory-induced Tet-R and Chl-R variants, the Tet/Chl -susceptible *E*. *coli* and *K*. *pneumoniae* strains which were designated as parental strains were exposed to successively increasing concentrations of Tet and Chl separately according to previously described method which was developed in our laboratory [11, 30]. Briefly, a portion of bacterial isolates grown on antibiotic-free Eosin Methylene Blue (EMB) agar were diluted in a sterile saline solution and were spread (3x 10^5^ colony-forming unit (CFU)/ml) on EMB agar containing the ½ and ¼ of MICs of Tet and Chl in parental isolates respectively and incubated at 37°C for 24-72hr. Three colonies per isolate were randomly selected from colonies grown on Tet/Chl containing plates and sub-cultured on EMB agar supplemented with previous (1X) and higher concentrations (1.2X-1.5X of previous concentration) of antibiotic and incubated at 37°C for 24-72hr. In each selection cycle, colonies were picked from media containing higher Tet/Chl concentration and used for the next selection cycle. This successive subculturing on EMB containing increasing concentrations of antibiotic continued until a ≥4 times increase in Tet and Chl MIC values was observed compared to initial values. To assess the stability of resistance phenotype obtained by *in-vitro* selection assay, all resistant isolates were sub-cultured on antibiotic-free culture media for 10 consecutive days followed by MIC determination.

### Antimicrobial susceptibility testing

Susceptibility of parental strains as well as respective Tet-R or Chl-R mutants to tetracycline, chloramphenicol, tigecycline and ciprofloxacin was determined by broth dilution method. Susceptibility to other antibiotics including ceftriaxone, ceftazidime, imipenem, nalidixic acid, doxycycline, gentamicin, and amikacin was determined using disc diffusion assay according to CLSI guidelines. Interpretation of the susceptibility testing results for all antibiotics (except for Tgc) was performed according to CLSI instructions. The Food and Drug Administration (FDA) and European Committee on Antimicrobial Susceptibility Testing (EUCAST) breakpoints issued for *Enterobacterales* were applied for interpretation of TGC susceptibility testing results in *K*. *pneumoniae* (susceptible ≤ 2 mg/L, intermediate = 4 mg/L, and resistant ≥ 8 mg/L) and *E*. *coli* (susceptible ≤0.5 mg/L, and resistant >0.5 mg/L) respectively. *Escherichia coli* ATCC 25922 was used as a quality-control strain for antimicrobial susceptibility testing.

### Analysis of genetic alterations of AcrAB regulators

In order to identify the molecular mechanisms mediating cross-resistance to Tgc after repeated exposure to Tet and Chl, the genetic alterations within AcrAB regulatory proteins were investigated. To this end, the nucleotide sequences of *acrR*, *ramR*, *soxR* genes in *K*. *pneumoniae* and *lon*, *marR*, *acrR* and *soxR* genes in *E*. *coli* were amplified using primers listed in Table 1 under the following PCR condition:1 cycle of 95°C for 10 min, 30 cycles of 95°C for 40 s (denaturation), 57°C (*acrR*_*K*. *pneumoniae*_, *marR*)/58°C (*ramR*, *acrR*_*E*.*coli*_)/ 59°C (*soxR*_*E*.*coli*_)/ 60°C (*lon*)/ 61°C (*soxR*_*K*. *pneumoniae*_) for 40 s (annealing), and 72°C for 40 s (extension) and a final extension of 72°C for 10 min. The amplified DNA fragments were separated by agarose gel electrophoresis and visualized under UV light.

**Table 1:** Nucleotide sequences of primers used in this study.

Primer name	Sequence (5ʹ to 3ʹ)	Product Size (bp)	Reference(s)
***K*. *pneumoniae***			
Sequencing			
soxR-F	GGCCTCTCTCAATTCTCGATTCG	613	This study
soxR-R	GCGAACCAGCGAGATAATGCG		
ramR-F-ext	TGGTCAGACGTGCCAAGATC	654	[11]
ramR-R-ext	CAGTGTTTCCGGCGTCATTAG		
ramR-F-int	GCAAGCGTTACTGGAAGCTG	515	
ramR-R-int	CAAAGCCAAGGGCGATAATCT		
acrR-F	GTAAAGTCATTAACCTATGGCACG	667	
acrR-R	TTAAGCTGACAAGCTCTCCG		
RT-qPCR			
acrB-F	CAGCTTAACGCCTCGATCATC	127	
acrB-R	CCAGCTCAATTTTGGCGACATC		
***E*. *coli***			
Sequencing			
marR-F	CAACTAATTACTTGCCAGGGCA	461	This study
marR-R	ACGGCAGGACTTTCTTAAGC		
acrR-F	CGTCGTGCTATGGTACATACATT	729	
acrR-R	GGAGTCAGATTCAGGGTTATTCG		This study
lon1-F	GTCGTGTCATCTGATTACCTGGC	1107	
lon1-R	GCAGAGGATCGGACCCTTGAT		
lon2-F	TCGACTGGATGGTACAGGTGC	1482	This study
lon2-R	CGCGAGGTCACTATTTTGCAGT		
soxR-F	CGTCAATCCATGCGATAAGATCC	671	This study
soxR-R	AAACCCTCCTGTGTACCAGC		
RT-qPCR			
acrB-F	TGGTCAGATGGTGCCGTTCTC	159	This study
acrB-R	GCCAGTTGTTCCATCAGCTCCA		
16S rRNA-F	ACCGTGAGACAGGTGCTGCAT	157	This study
16S rRNA-R	GTCATCCCCACCTTCCTCCAG		

The PCR amplicons were sequenced by Sanger sequencing and the nucleotide sequences of the studied genes in Tet-R and Chl-R mutants were analyzed by ChromasPro software (Version 2.1.3) and compared to nucleotide sequences of their respective ancestor strains as well as *K*. *pneumoniae* ATCC 13883 and *E*. *coli* K12 for identification of mutations using BLAST found on the National Center for Biotechnology Information (NCBI) website.

### Transcript analysis by reverse transcription quantitative polymerase chain reaction (RT-qPCR)

The total RNA from all mutants and parental cells was harvested using AddPrepTotal RNA Extraction Kit (AddBio, Korea). The complementary DNA (cDNA) was synthesized from 500ng of DNase I (Thermo Scientific)-treated total RNA using AddScript cDNA synthesis kit (AddBio, Korea) according to the manufacturer’s instructions. Real-time PCR amplification was performed using a RealQ Plus 2x Master Mix Green (Ampliqon) on an Eco Real-Time PCR system (Illumina) under the following condition: 1 cycle of 95°C for 10 min, 45 cycles of 95°C for 15 s, 60°C for 30 s and 72°C for 25 s. The relative gene expression levels of *acrB* were calculated using the 2^−ΔΔCT^ with 16S *rRNA* as internal control for both *E*. *coli* and *K*. *pneumoniae* isolates. The expression levels of *acrB* among mutants were compared with those of parental strains. The oligonucleotide sequences used for amplification of *acrB*, and *16s rRNA* fragments are listed in Table 1. Statistical analyses were performed using GraphPad Prism (9.0.0) (unpaired *t*-test). P values of statistical significance were illustrated as follows: * p≤0.05; ** p ≤ 0.01; *** p ≤0.001; n.s. represents non-significant differences (P>0.05).

## Results

### Bacterial isolates and drug susceptibility testing

In order to identify possible factors contributing to Tgc resistance emergence in the absence of Tgc selective pressure, *E*. *coli* and *K*. *pneumoniae* isolates were exposed to successively increasing concentrations of Tet and Chl. A total of 12 variants including 6 Tet-R (3 *E*. *coli*, 3 *K*. *pneumoniae*) and 6 Chl-R (3 *E*. *coli*, 3 *K*. *pneumoniae*) were obtained after about 15 to 40 selection cycles in the presence of antibiotic.

**Fig 1:**
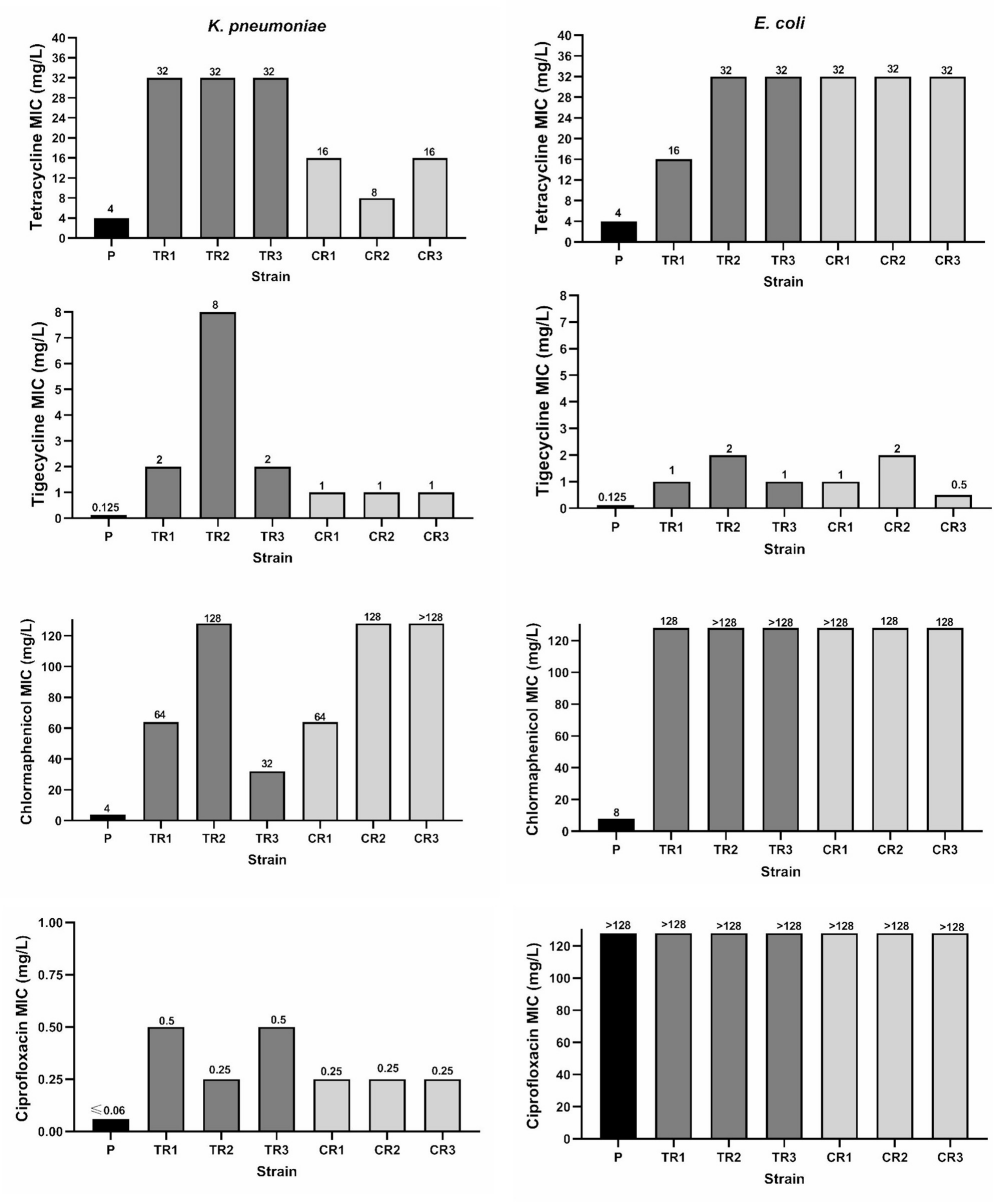
**MIC changes observed after *in vitro* exposure of *K*. *pneumoniae* (left) and *E*. *coli* (right) to tetracycline and chloramphenicol.** P, parent strain; TR1-3, tetracycline-selected mutant 1–3; CR1-3, chloramphenicol-selected mutant1-3.

While in Tet-R mutants, the *in vitro* selection assay in the presence of Tet, rose tetracycline MICs 4–8 times in both studied species compared to parental isolates, tigecycline MICs in *K*. *pneumoniae* and *E*. *coli*, increased 16–64, and 8–16 times, and chloramphenicol MICs increased 8–32 and ≥16 times respectively. On the other hand, Chl-exposed mutants showed 16 to >32(*K*. *pneumoniae*) and ≥16 times (*E*. *coli*) increase in Chl MICs compared to their respective ancestors, and revealed MIC increase of 8 and 4–16 times for Tgc and 2–4 and 8 times for Tet in *K*. *pneumoniae* and *E*. *coli* mutants respectively. Moreover, both Tet and Chl exposure resulted in at least 4 times increase in ciprofloxacin MICs in *K*. *pneumoniae* parental isolate (Fig 1). In general, both tetracycline and chloramphenicol exposure resulted in resistance/decreased susceptibility to not only tigecycline but also to other members of tetracycline family and quinolones. However, susceptibility to studied aminoglycosides and β-lactams remained unchanged in both groups of antibiotic-resistant variants as determined by disk diffusion test (Table 2). The successive passage of resistant bacteria in antibiotic-free culture media following resistance-induction assay, was not associated with decrease in Tet, Chl and Tgc MICs indicating that the obtained resistant phenotype was stable.

**Table 2:** Antimicrobial susceptibility testing results for *in vitro* selected tetracycline and chloramphenicol-resistant strains as well as their respective parental isolates, determined by broth dilution and disc diffusion methods.

	Broth dilution							Disc diffusion						
	(Tet-R)				(Chl-R)				(Tet-R)			(Chl-R)		
	Tet	Chl	Tgc	Cip	Tet	Chl	Tgc	Cip	S	I	R	S	I	R
Strain	(mg/L)				(mg/L)									
*K*. *pneumoniae*														
**KP-p**	4	4	0.125	≤0.06	4	4	0.125	≤0.06	NA, DO, GM, AK, CR, CZ, ME			NA, DO, GM, AK, CR, CZ, ME		
**KP-m1**	32	64	2	0.5	16	64	1	0.25	CM, AK, CR, CZ, ME		**NA, DO**	GM, AK, CR, CZ, ME		**NA, DO**
**KP-m2**	32	128	8	0.25	8	128	1	0.25	GM, AK, CR, CZ, ME		**NA, DO**	GM, AK, CR, CZ ME	**DO**	**NA**
**KP-m3**	32	32	2	0.5	16	>128	1	0.25	GM, AK, CR, CZ, ME		**NA, DO**	GM, AK, CR, CZ ME		**DO, NA**
***E*. *coli***														
**EC-p**	4	8	0.125	-^a^	4	8	0.125	_	DO, AK, ME		NA, CR, CZ, GM	DO, AK, ME		NA, CR, CZ, GM
**EC-m1**	16	128	1	-	32	>128	1	_	AK, ME	**DO**	NA, CR, CZ, CM	AK, ME	**DO**	NA, CR, CZ, GM,
**EC-m2**	32	>128	2	-	32	128	2	_	AK, ME		NA, CR, CZ, GM, **DO**	AK, ME		**DO,** NA, CR, CZ, GM,
**EC-m3**	32	>128	1	-	32	128	0.5	_	AK, ME	**DO**	NA, CR, CZ, GM	AK, ME	**DO**	NA, CR, CZ, GM,

**Tet-R/Chl-R:** *in vitro-*selected mutants developed under selective pressure of Tet/Chl

**KP-p,** *K*. *pneumoniae* parent strain; **EC-p**, *E*. *coli* parent strain

**Tgc**,Tigecycline, **Tet**, Tetracycline, **Chl**,Chloramphenicol, **Cip**,Ciprofloxacin. **CR**, ceftriaxone; **CZ**: ceftazidime, **ME**: meropenem, **AK**, amikacin; **GM**: gentamicin; **DO**, doxycycline, **NA**: nalidixic acid

^a^ -, high level ciprofloxacin resistance (MIC>128mg/L)

### Sequence analysis of AcrAB regulatory proteins

Since the increased efflux of antibiotic was suspected to be involved in cross- resistance to similar as well as structurally dissimilar agents, we determined the nucleotide sequences of genes coding for major regulators of AcrAB system in both bacteria including *ramR*, *acrR* and *soxR* in *K*. *pneumoniae* and *marR*, *lon*, *acrR* and *soxR* in *E*. *coli* isolates. The comparison of nucleotide sequences of studied genes between *in vitro*-selected mutants of *E*. *coli* and their ancestral isolate revealed several MarR changes including L33R, A70T and G15S amino acid substitutions. In *lon*, though, we found a L630F change and frameshift mutation (due to deletion of one nucleotide) presumably inactivating Lon protein. The latter change was co-harbored by MarR A70T change in EC-TR2 strain (Table 3). Analysis of *acrR* and *soxR* genes showed no alteration in comparison with parental isolates. However, compared to reference strains *E*. *coli* K12, we identified MarR G103S, Y137H, SoxR G74R amino acid changes and *acrR* inactivation due to insertion of *IS1*-like family (768 bp,) at position +147/148 in *E*. *coli* parental strain as well as their respective variants.

**Table 3:** Genetic alterations in AcrAB regulatory proteins in *in vitro* selected Tet-R or Chl-R-mutants compared to that in parental strain.

*K*. *pneumoniae*	Fold change in MIC (times)^a^			RamR	Selection cycles	*E*. *coli*	Fold change in MIC (times)			MarR	Lon	Selection cycles
	tgc	tet	chl				tgc	tet	chl			
**Tetracycline-resistant mutants**						**Tetracycline-resistant mutants** ^f^						
** KP-TR1**	16	8	16	- ^b^	40	**EC- TR1**	8	4	16	L33R	-	40
** KP-TR2**	64	8	32	Dup of 8nt (position133-140)^C^ frame shift	39	**EC- TR2**	16	8	>16	A70T	ΔG1615^g^ frameshift	37
** KP-TR3**	16	8	8	Del of 5nt (C86-G90) frame shift ^d^	39	**EC- TR3**	8	8	>16	-	-	37
**Chloramphenicol-resistant mutants**						**Chloramphenicol -resistant mutants**						
** KP-CR1**	8	4	16	Δ*ramR* locus ^e^	25	**EC-CR1**	8	8	>16	-	L630F	16
** KP-CR2**	8	2	32	-	16	**EC-CR2**	16	8	16	G15S	-	15
** KP-CR3**	8	4	>32	-	27	**EC-CR3**	4	8	16	-	-	25

^a^ the amount of MIC value increase in the mutants compared to parental isolates

^b^ -, unaltered values compared to parental strain

^C^ Duplication of TTTCGCTA

^d^ Deletion of CGACG

^e^Δr*amR* locus, not amplifiable with all primers used in this study

^f^MarR G103S, Y137H, SoxR G74R amino acid changes and *acrR* inactivation due to insertion of IS1-like family (768 bp,) at position +147/148 were found among parental strain and respective decedents of *E*. *coli*

^g^ deletion of guanine at position 1615 of *lon* gene

On the other hand, among the three different genes studied in *K*. *pneumoniae* mutants, *ramR* was the only altered locus which was found to be inactivated (due to frameshift mutations) or possibly deleted. In the latter case, no PCR product was obtained by amplification of *ramR* coding sequence as well as its flanking regions, using two different pair of primers (ramR-int & ramR-ext, (Table 1)) which raised the possibility of *ramR* locus deletion in this isolate (Table 3). The nucleotide sequences of the studied genes have been deposited at GenBank nucleotide sequence database under the following accession numbers: PP990503- PP990515 and PQ106660.

### Transcriptional analysis of *acrB*

**Fig 2:**
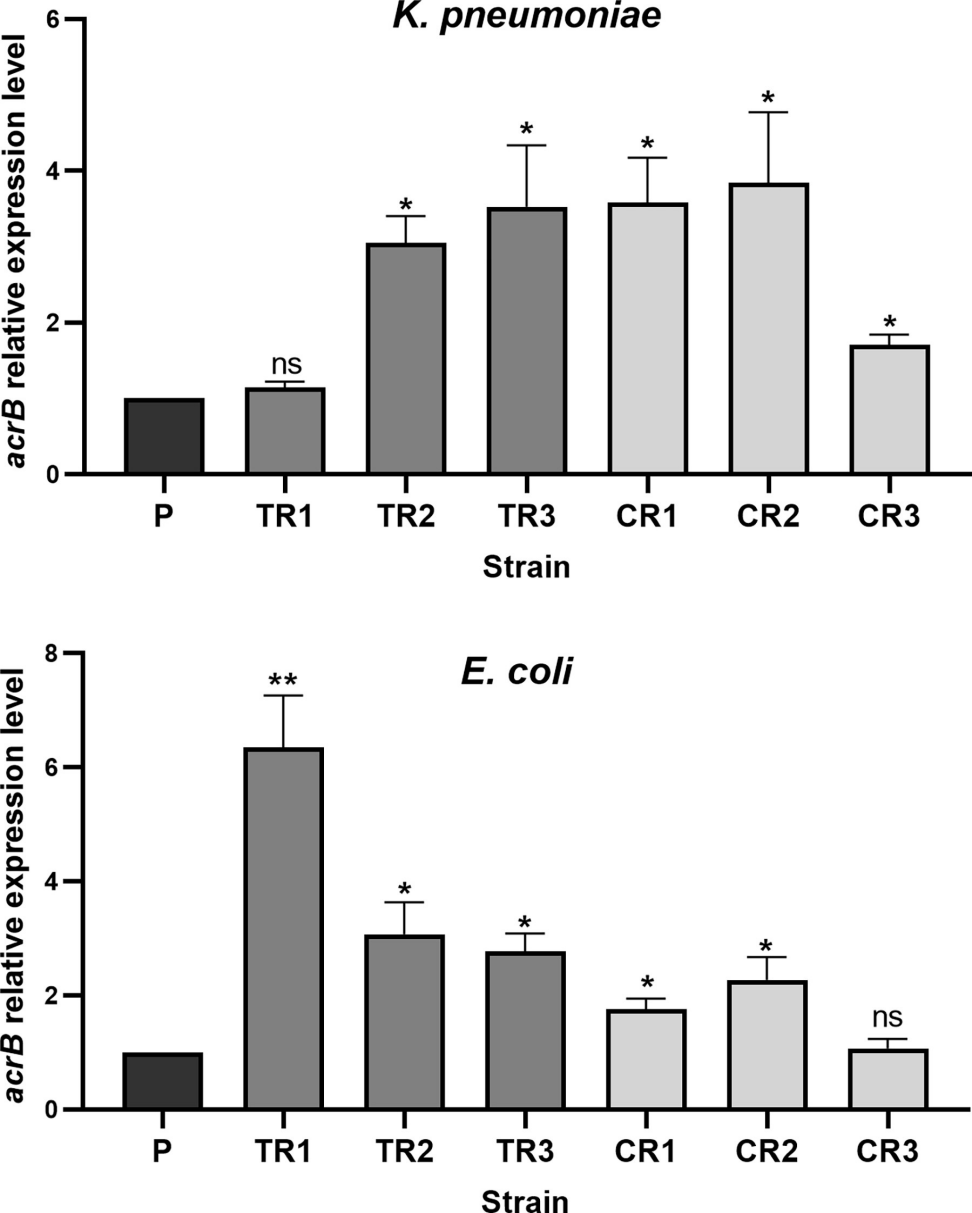
Gene expression analysis of *acrB*. The expression level of *acrB* was measured by RT-qPCR and was normalized to *16S rRNA*. Each value represents the mean ± standard deviation for three independent experiments. * p ≤0.05; ** p ≤ 0.01; ns, non-significant p>0.05.

To prob the molecular basis of multiple-antibiotic resistance (MAR) phenotype found among laboratory-induced Tet-R and Chl-R strains, we measured the expression level of *acrB* gene using RT-qPCR analysis. According to results, 83% of *E*. *coli* (n = 5) and *K*. *pneumoniae* (n = 5) strains revealed ∼1.7 to 6.4-fold and 1.6 to 3.8-fold increase in the expression of *acrB* compared to their respective parental strains respectively. From five strains lacking genetic alterations in the studied genes, three (2 *K*. *pneumoniae* (KP-CR2, KP-CR3) and 1 *E*. *coli* (EC-TR3)), displayed an overexpressed *acrB* with the remaining two strains (KP-TR1, EC-CR3) showing unaltered *acrB* expression levels (Fig 2).

## Discussion

Increased incidence of antimicrobial resistance poses a serious global threat to human health as it compromises our ability to treat common infectious diseases such as urinary or respiratory tract infections, typhoid fever, as well as more severe and potentially fatal illnesses, including bacteremia and pneumonia resulting in severe illness, prolonged hospitalizations, higher healthcare costs, disability and even death [31]. It can also impact the health of animals, productivity in farms and food safety. The agricultural use of human antibiotics as growth promoters or as prophylactic agents in farm animals are considered as strong drivers of the local and global distribution of antimicrobial resistance [32]. Livestock, poultry and other avian species have been found as important reservoirs for the transmission of antimicrobial resistance genes (ARGs) [33]. These ARG- harboring bacteria can be easily transmitted between humans, animals, and the associated environments. Tetracycline antibiotics (including chlortetracycline, oxytetracycline, tetracycline and doxycycline,) account for 35.3% of the total antimicrobials used in animals in 2016 [34]. The extensive use of this family of antibiotics has resulted in development of Tet resistant bacteria in animal and human commensals as well as environmental bacterial. Tigecycline, a third-generation tetracycline antibiotic belongs to glycylcyclines which are derived from the semisynthetic minocycline, in an effort to overcome common Tet resistance mechanisms. Despite its promising activity against a variety of Gram-positive and negative bacteria Tgc has never been allowed to be used in animal husbandry anywhere around the world [33, 35]. However, recent surveillance studies have reported higher prevalence of tigecycline resistance genes in animals than in humans [34, 36, 37]. Recently we reported isolation of several Tgc-R-KP isolates from healthy broilers. Overexpression of AcrAB efflux pump driven by several mutations in genes coding for AcrAB regulatory system such as *acrR* and *ramR* was identified as possible underlying resistance mechanism in these animal-derived Tgc-R -KP isolates [11]. In the current work we investigated the association between increased exposure to Tet or Chl and Tgc susceptibility changes. Our results revealed that excessive exposure to both antibiotics significantly decreased susceptibility of tetracycline and chloramphenicol-selected mutants of *E*. *coli* (4–16 time) and *K*. *pneumoniae* (8–64 times) to tigecycline. Therefore, it is concluded that consumption of first-generation tetracyclines and phenicols in animals can provide enough selective pressure for development of mutants which are not only resistant to these antibiotics but also to tigecycline a clinically important last-resort agent. The positive correlation between animal florfenicol use and accumulation of tigecycline resistance genes (TRGs) have been described in a recent study [34].

One of the major mechanisms contributing to decreased susceptibility to antimicrobials in bacteria involves reduced intracellular concentration of antibiotic conferred by overexpression of efflux pump genes and/or porin deficiencies. The AcrAB-TolC system is among the best characterized efflux pumps in *Enterobacterales*. The substrate range of AcrB, a transporter of the RND type is very diverse and includes a variety of agents such as chloramphenicol, fluoroquinolones, tetracycline, β-lactams, erythromycin, rifampicin, disinfectants, dyes, and organic solvents [38]. This pump is regulated by local repressor AcrR and transcriptional activators RamA in *K*. *pneumoniae* [39] or by local repressor AcrR, and global transcriptional activator, MarA in *E*. *coli* [40]. The biological levels of MarA and RamA seem to be the critical determinants of antibiotic resistance and repressors such as MarR in *E*. *coli* and RamR in *K*. *pneumoniae* represses the transcription of genes coding for these activators under normal conditions. MarR/RamR mutations can result in expression of *marA* or *ramA* respectively which in turn promote expression of AcrAB efflux pump [39, 40]. MarA in *E*. *coli* also plays an important role in promoting expression of *micF* locus coding for small inhibitory RNA that down-regulates the outer membrane porin OmpF by posttranscriptional decrease in *ompF* mRNA[41]. Indeed, in this case increased efflux can be coupled to decreased permeability as a result of increased level of MarA. The MarA levels can be regulated post-translationally via rapid degradation by ATP-dependent Lon protease in *E*. *coli* [19]. Previous studies have reported *marR*, *soxR*, *lon*, *acrR* mutations in numerous highly antibiotic-resistant isolates of *E*. *coli* [21, 42].

According to substrate range of AcrAB we suggested that cross-resistance observed for tigecycline after tetracycline or chloramphenicol exposure could be attributed to overexpression of AcrAB efflux system. Therefore, we determined the nucleotide sequences of genes coding for major regulators of AcrAB and identified several genetic alterations only in 58.3% (n = 7) of laboratory induced resistant strains. The identified changes included, frameshift mutations in *ramR* and possibly *ramR* locus deletion (in *K*. *pneumoniae*), MarR L33R, A70T, G15S amino acid substitutions and *lon* frameshift mutation or L630F change (in *E*. *coli*) which resulted in elevated *acrB* expression levels. However, no genetic alteration was found among 5 resistant isolates from which some revealed overexpressed *acrB* Due to regulation of AcrAB by a complex network of proteins, alterations in other genes involved in AcrAB expression (such as *rob*) [43, 44] can explain upregulation of this efflux pump in resistant strains lacking genetic changes in the studied loci. Similarly, Ghisalberti et al. reported chloramphenicol exposure as an inducer of MDR phenotype in *in vitro-*selected chloramphenicol-resistant variants of *Klebsiella aerogenes* (formerly known as *Enterobacter aerogenes*) which revealed increased expression of AcrAB–TolC efflux system but lacked any alterations in *acrR* and *ramR* genes or Omp36 expression levels [45]. Similarly, in our previous study we could not identify AcrR or RamR alterations in some laboratory induced or animal originated Tgc-R-KP isolates with overexpressed *acrB* gene [11]. Contribution of other efflux systems or non-efflux-based resistance determinants (such as lack of porins in outer membrane) [45, 46], may explain the evolution of MAR phenotype in resistant strains with unaltered (strains lacking changes in the studied genes) or slightly increased *acrB* expression levels (in *E*. *coli* CR1, considering the reported low effect of *lon* mutations alone on gaining drug resistance [42, 47]). Previous studies have reported mutations in LPS biosynthesis pathway in relation to tigecycline resistance among Tgc-selected mutants of *E*. *coli* [20, 21]. Cohen et. al reported association between quinolone resistance development and MarA-dependent OmpF reduction among tetracycline or chloramphenicol- selected mutants of *E*. *coli* [48].

In addition to genetic changes identified among *in vitro-*selected resistant strains, we detected some amino acid substitutions among Tet/Chl susceptible parental *E*. *coli* strain including G103S combined withY137H in MarR, G74R amino acid substitution in SoxR and *acrR* inactivation due to insertion of *IS1*. The G103S, Y137H amino acid changes are known not to affect repressor activity of MarR [49, 50]. Despite AcrR repressor inactivation by *IS1*, the parental strain remined susceptible to, tetracycline, doxycycline, chloramphenicol, amikacin and imipenem. According to whole-genome sequencing data (which were available for this strain from our previous study) this isolate harbored *bla*_CTX-M-15_, *aadA5*+*aac(3)-IIa* genes and mutations in QRDR region (GyrA S83L, D87N and ParC S80I, E84V) which are known to be involved in resistance to some β-lactams, aminoglycosides and quinolones respectively. Therefore, the contribution of *acrR* inactivation to MAR phenotype observed among this strain harboring other resistance conferring determinants requires further experimental studies.

Tet and Chl resistance induction in our study resulted in development of a multi-drug resistance phenotype being characterized with resistance/decreased susceptibility to not only tigecycline but also other tetracyclines, chloramphenicol and quinolones. However, susceptibility to studied aminoglycosides and β-lactams did not change. Similarly, a previous study linked chloramphenicol resistance to an active efflux of this antibiotic and reported cross-resistance to fluoroquinolones and tetracycline, but not to aminoglycoside or b-lactam antibiotics [45, 51].

Altogether our results provided the first *in vitro-*based evidences that tetracyclines and phenicols excessive exposure can contribute to the development of bacteria with resistance or decreased susceptibility to tigecycline which have the potential of being spread in an animal-human–environment cycle. This finding indicates that imprudent use of tetracyclines and phenicols in animals can be considered as an important mediator of emergence of bacteria which are resistant to an antibiotic used for treating deadly human infections caused by extensively drug-resistant superbugs. Farm-based studies including several groups of animals feeding on tetracycline or florfenicol containing meals and comparing them with those which are not previously exposed to these antibiotics are required to prove an *in vivo* effect of exposure to these agents on tgc resistance development among the studied animals’ gut microbiome. Moreover, further experimental work would be required to determine the role of other cross-resistance conferring mechanisms such as porin deficiencies/reduction in tetracycline/chloramphenicol-selected mutants.
